# Adenocarcinoma of the Lung Masquerading As Invasive Pulmonary Aspergillosis in an Elderly Lady: A Diagnostic Challenge

**DOI:** 10.7759/cureus.53345

**Published:** 2024-01-31

**Authors:** Ramyashree N Reddy, Nandakishore Baikunje, Giridhar Belur, Nandu Nair

**Affiliations:** 1 Internal Medicine, KS Hegde Medical Academy, Mangaluru, IND; 2 Pulmonary Medicine, KS Hegde Medical Academy, Mangaluru, IND

**Keywords:** bronchogenic adenocarcinoma, occult disease, elderly population, lung cancer, aspergillosis

## Abstract

A 59-year-old hypertensive woman presented with a year-long history of cough, expectoration, and progressive breathlessness, recently complicated by hemoptysis and significant weight loss. Initial investigations, including a chest x-ray and contrast-enhanced computed tomography (CECT) of the thorax, suggested an infective pathology. Despite negative bacterial, fungal, and tuberculosis cultures, elevated bronchoalveolar lavage (BAL) galactomannan and serum Aspergillus-specific IgG levels led to a diagnosis of invasive pulmonary aspergillosis (IPA), and antifungal treatment commenced. The patient's initial response was positive; however, symptoms recurred three months later. Further investigations revealed adenocarcinoma, confirmed by cytology from a thoracentesis. The patient, a non-smoker, began targeted therapy with tyrosine kinase inhibitors but declined further diagnostic evaluation. Despite the poor prognosis and palliative care options, the patient opted for discharge to home care. This case underscores the complexity of diagnosing lung pathologies and the importance of considering alternative diagnoses in persistent respiratory symptoms.

## Introduction

Adenocarcinoma is the most common subtype of lung cancer [1]. Fungal pneumonia can present with similar symptoms and radiological findings to lung cancer [2]. The diagnostic challenges are further complicated by the fact that both conditions can occur concurrently or in succession. A thorough clinical evaluation, imaging studies, and biopsy may be necessary to establish a definitive diagnosis. Adenocarcinoma is typically treated with a combination of surgery, radiation therapy, and chemotherapy while fungal pneumonia is treated with antifungal medications. This case highlights the diagnostic challenges associated with respiratory symptoms and the need for careful evaluation and monitoring of elderly patients. It also underscores the importance of considering multiple differential diagnoses, as well as the need for ongoing assessment and follow-up, even after an initial diagnosis and treatment. This case may be of interest to clinicians and researchers in the fields of respiratory medicine, oncology, and infectious diseases, as well as those interested in the diagnostic challenges associated with complex medical cases.

## Case presentation

**Figure 1:**
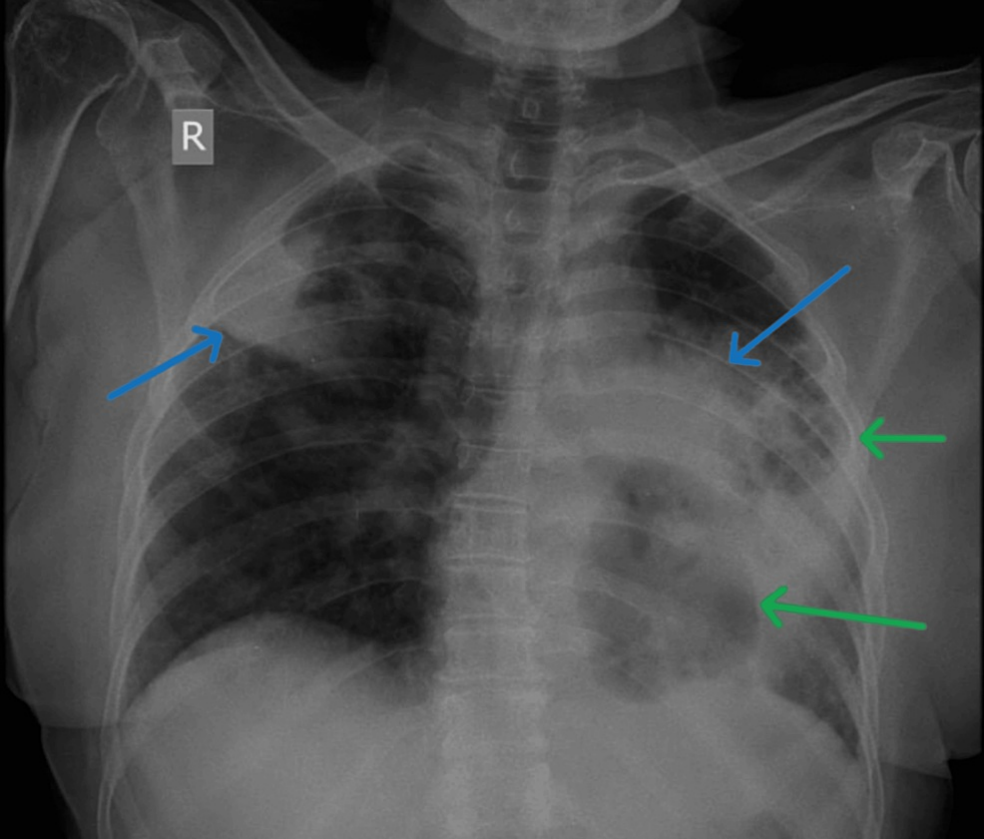
Chest x-ray showing consolidative changes in the right upper zone and left mid zone (blue arrows) with cavitary changes in the left mid and lower zones (green arrows)

A 59-year-old woman, known hypertensive on medication came with complaints of cough with expectoration, breathlessness for one year aggravated for one month, and hemoptysis for one week. The cough was insidious in onset, occurred in bouts, was gradually progressive, associated with mucoid expectoration, scanty in amount, white in color, non-blood stained initially, and non-foul smelling. Breathlessness was also insidious in onset, starting as breathlessness on exertion (Modified Medical Research Council (MMRC) grade 0) and gradually progressing to breathlessness at rest (MMRC grade 4). There is no increase with change in position, and no symptoms suggestive of paroxysmal nocturnal dyspnea (PND). She has no history of fever. She noticed blood in her sputum one week back, massive (500mL/day). She reports a weight loss of 10kg in one year. Routine blood investigations showed elevated counts. Bleeding parameters were normal. She was started on oral Azithromycin and hemostatic agents. Chest x-ray (CXR) showed consolidative changes in the right upper and left mid zone with cavitatory changes in the left mid zone (Figure 1).

**Figure 2:**
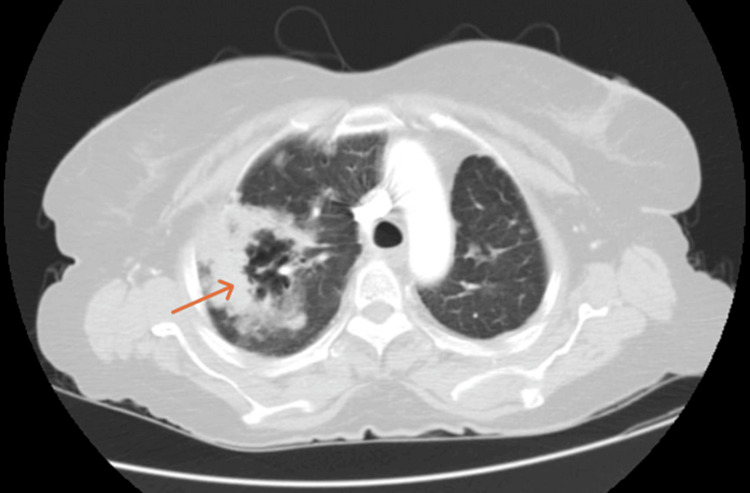
Contrast-enhanced computed tomography of the thorax showing cavitary lesion in the right upper lobe with perilesional consolidative changes (orange arrow)

**Figure 3:**
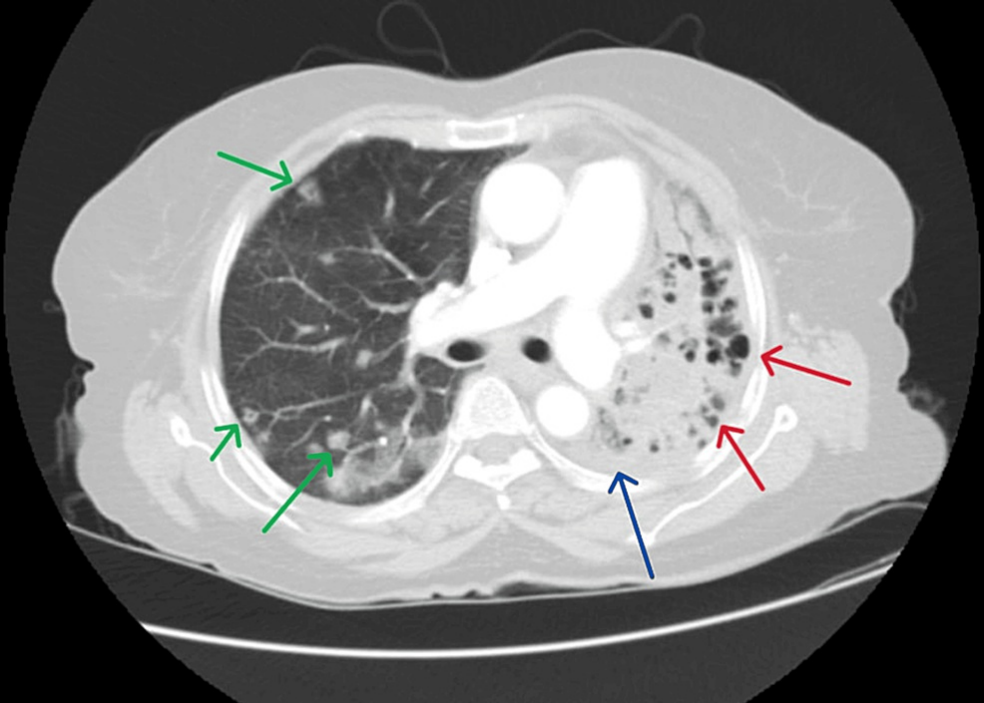
Contrast-enhanced computed tomography of the thorax showing multiple nodular lesions on the right side (green arrows) and cavitary lesions with dilated airways on the left (red arrows) with mild pleural effusion (blue arrow)

**Figure 4:**
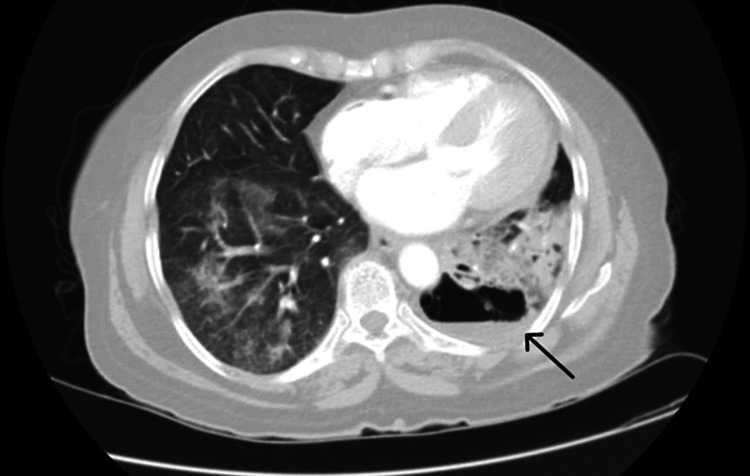
Contrast-enhanced computed tomography of the thorax showing consolidative changes with a lung abscess in the left lower lobe (black arrow)

A contrast-enhanced computed tomography (CECT) of the thorax was done which showed “Bilateral patchy and confluent airspace consolidation, multiple nodular lesions of both lungs, cavitatory lesions of bilateral lung fields with dilated airways with mild left-sided pleural effusion, altogether suggestive of an infective pathology” (Figures 2-4).

**Figure 5:**
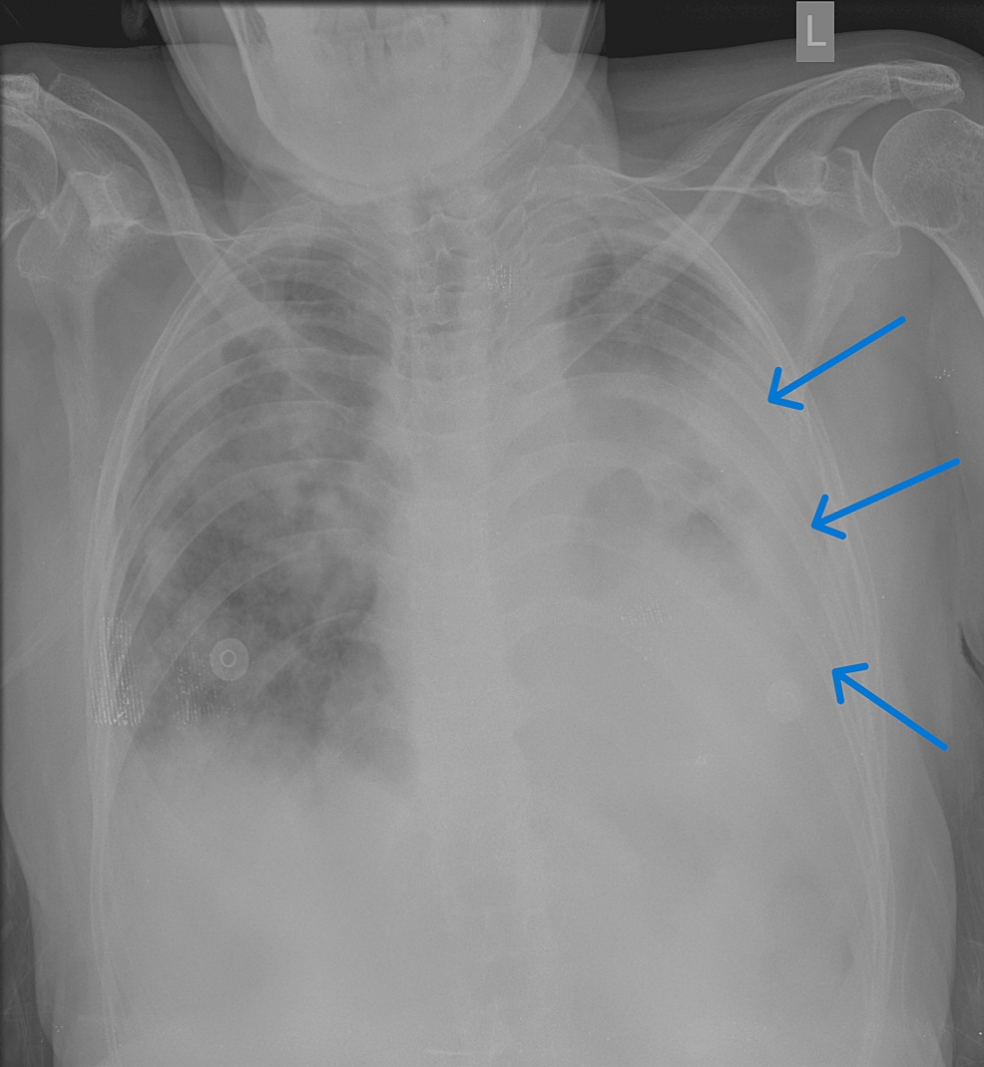
Chest x-ray during the subsequent admission showing an increase in the opacity on the left hemithorax (blue arrows)

Sputum was sent for bacterial culture, fungal culture, and GeneXpert for tuberculosis which were all negative. Ultrasound (USG) of the thorax was done which showed that the effusion was mild and not amenable to tapping. Connective tissue disease screening was done and was negative. She was hence planned for a bronchoscopy which showed only secretions and no endobronchial lesions. A bronchoalveolar lavage (BAL) was taken and BAL cytology showed “mixed inflammatory cell infiltrate.” Since she was not responding to antibiotics and cultures were sterile, BAL galactomannan was sent and was significantly elevated. Simultaneously, serum Aspergillus-specific IgG was also sent and found to be positive. Hence a diagnosis of invasive pulmonary aspergillosis (IPA) was made, and she was started on appropriate antifungals. Following this, the patient felt subjectively better and was discharged. Three months later, she presented back to our outpatient department (OPD) with increased breathlessness and cough aggravated for 1.5 months and hemoptysis for one week. An alternative diagnosis was considered. Repeat CXR showed an increase in the opacity on the left side, almost causing a “white-out” of the left lung field (Figure 5).

**Figure 6:**
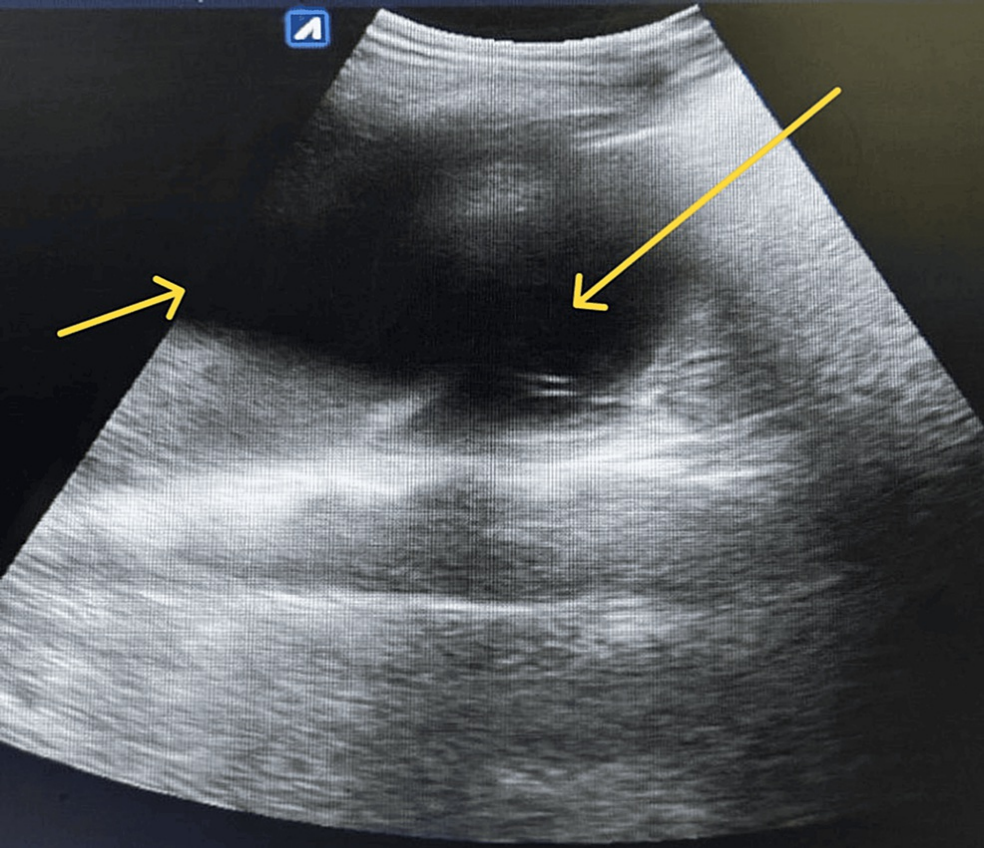
Ultrasonography of the left hemithorax showing moderate pleural effusion (yellow arrows)

Repeat USG thorax was done which showed moderate pleural effusion (Figure 6).

Diagnostic + therapeutic thoracentesis was done during which hemorrhagic fluid of 500mL was drained and sent for analysis. It showed an exudative picture with cytology showing malignant cells suggestive of adenocarcinoma. A medical oncology opinion was taken following which she was advised positron emission tomography-computed tomography (PET-CT) for further evaluation for which she was not willing. Owing to her non-smoking status with cytology suggestive of adenocarcinoma, she was started on tyrosine kinase inhibitors as part of targeted therapy. The patient and her attendees were explained the poor prognosis and options for palliation but wanted to take her home. She was hence discharged.

## Discussion

Adenocarcinoma is the most common subtype of lung cancer, accounting for approximately 40% of all lung cancers [1]. It arises from the glandular cells that line the lung tissue and is often aggressive in nature and tends to metastasize. The prognosis of this variant of lung cancer is relatively better compared to other variants due to the effectiveness of tyrosine kinase inhibitors which suppress the tumor activity through molecular methods. Fungal pneumonia, on the other hand, is a respiratory infection caused by various species of fungi, including Aspergillus, Histoplasma, and Cryptococcus. Although these two conditions are distinct, they can present with similar symptoms and radiological findings, leading to diagnostic challenges [2]. Adenocarcinoma of the lung can present with a range of symptoms, including cough, breathlessness, chest pain, weight loss, and fatigue. These symptoms can also occur in fungal pneumonia, particularly in cases of invasive fungal infections. Invasive aspergillosis, for example, can cause cough, dyspnea, hemoptysis, and pleuritic chest pain. Fungal pneumonia can also be associated with fever, chills, night sweats, and malaise, which can be non-specific and overlap with the symptoms of lung cancer. Radiological imaging can also be similar in both conditions. Adenocarcinoma of the lung can manifest as a mass or nodule on a CXR or computed tomography (CT) scan. Fungal pneumonia can also present with nodular infiltrates or consolidation on chest imaging, which can mimic lung cancer. In some cases, fungal pneumonia can cause cavitary lesions, which can also occur in lung cancer. The diagnostic challenges associated with adenocarcinoma of the lung and fungal pneumonia are compounded by the fact that they can occur concurrently or in succession. Patients with a history of lung cancer are at increased risk of developing fungal infections, particularly if they have undergone chemotherapy or radiation therapy. In some cases, fungal infections can precede the diagnosis of lung cancer, leading to delays in diagnosis and treatment. Similarly, lung cancer can also present with fungal infections, further complicating the diagnostic process [2].

Distinguishing between the two conditions requires a thorough clinical evaluation, including a detailed medical history, physical examination, and laboratory investigations. Imaging studies, including CXRs, CT scans, and positron emission tomography (PET) scans, can also help identify the location and extent of the disease. However, these tests are not always conclusive, and a biopsy or other invasive procedures may be necessary to establish a definitive diagnosis [3]. BAL galactomannan can be falsely elevated when the patient has had exposure to beta-lactam antibiotics. There are also reports of “plasmalyte” and IVIG both causing falsely elevated BAL galactomannan. As for serum Aspergillus-specific IgG, it can be falsely elevated if the patient had a previous infection with Aspergillus or with non-aspergillus mycoses. The treatment of adenocarcinoma of the lung and fungal pneumonia varies depending on the stage and severity of the disease. Adenocarcinoma of the lung is typically treated with a combination of surgery, radiation therapy, and chemotherapy, depending on the stage of the cancer. Fungal pneumonia, on the other hand, is treated with antifungal medications such as fluconazole, voriconazole, or amphotericin B, depending on the type and severity of the infection.

Wu et al. described four patients in their case series “pulmonary solid tumor coexisting with pulmonary aspergillosis” which included three elderly women and one old man who were diagnosed with both pulmonary malignancies as well as pulmonary aspergillosis. They concluded that the patients may have been immunodeficient due to the tumor and were thus more susceptible to the fungal infection [2] Nilsson et al. reported two cases of endobronchial pulmonary carcinoid with superimposed Aspergillus colonization. They reviewed the literature on 35 cases of non-carcinoid pulmonary carcinoma with superimposed aspergillosis. Among them, 28 were cavitary and seven were non-cavitary. They revealed that endobronchial cancer hidden under a cover of fungi, fibrin, and tissue debris will be missed on a superficial biopsy [4]. Malik et al. reported the prevalence of aspergillosis among 42 bronchogenic carcinoma patients in India as 14.2% [5].

Sheikh et al. detailed the case of a 32-year-old Nepalese gentleman, an ex-smoker and air conditioner technician, who was admitted with a week-long history of intermittent hemoptysis and mild chest discomfort, without any significant past medical history. Initial assessments, including physical examination and CXR, revealed no significant abnormalities apart from an irregular opacity in the right mid-zone of the lung. Routine laboratory tests were within normal limits, and screenings for connective tissue diseases and antineutrophil cytoplasmic antibodies were negative. Despite negative results for tuberculosis in sputum samples and a chest CT scan indicating a cavitary lesion in the left upper lobe suggestive of aspergilloma, subsequent bronchoscopy findings were unremarkable. The initial diagnosis of aspergilloma led to treatment with voriconazole and regular follow-ups. However, the patient's condition did not improve, as indicated by persistent hemoptysis and a follow-up CT scan showing worsening of the cavitary lesion with increased soft tissue component and more prominent ground-glass attenuation. Further investigation through a repeat bronchoscopy revealed mild inflammatory changes in the left upper lobe and growth of *Aspergillus nidulans* and *Candida parapsilosis* in bronchial wash samples. The worsening clinical picture and imaging findings prompted a referral to thoracic surgery. A left upper lobectomy performed on the patient led to the unexpected diagnosis of invasive adenosquamous carcinoma, as confirmed by histopathological examination. A subsequent PET scan showed no evidence of distant metastasis. The lung cancer multidisciplinary team proposed additional surgical resection or radical radiotherapy to ensure clear margins, offering the patient a choice in his treatment plan. This case underscores the complexities and challenges in diagnosing lung pathologies, highlighting the potential for initial misdiagnosis and the importance of thorough investigation and multidisciplinary collaboration in patient management [6].

Our case highlights the challenges associated with the differential diagnosis of respiratory symptoms that can occur in both IPA and lung cancer. The initial diagnosis of IPA was based on imaging studies and elevated fungal markers. However, the persistence and worsening of symptoms despite appropriate treatment raised the possibility of an alternative diagnosis. The increase in her effusion rightly prompted us to suspect an alternative diagnosis for which we did a thoracentesis which ultimately led to the diagnosis of adenocarcinoma. Further, although squamous cell carcinoma is typically associated with cavitary lesions in lung cancer, this case demonstrates that adenocarcinoma can also present as lung cavities.

## Conclusions

This case report underscores the importance of considering multiple differential diagnoses, as well as the need for ongoing assessment and follow-up, even after an initial diagnosis and treatment. High suspicion should be kept for malignancy whenever an elderly patient presents with non-resolving symptoms. Lung cancer may present as a cavitary disease of the lung and should be considered once infective pathologies are ruled out.
